# Pathogenic synergy: dysfunctional mitochondria and neuroinflammation in neurodegenerative diseases associated with aging

**DOI:** 10.3389/fragi.2025.1615764

**Published:** 2025-08-05

**Authors:** Shalini Mani, Samiksha Wasnik, Chesta Shandilya, Vidushi Srivastava, Saboor Khan, Keshav K. Singh

**Affiliations:** ^1^ Department of Biotechnology, Centre for Emerging Diseases, Jaypee Institute of Information Technology, Noida, India; ^2^ Division of Regenerative Medicine, Department of Medicine, Loma Linda University, Loma Linda, CA, United States; ^3^ Departments of Genetics, Dermatology, and Pathology, Heersink School of Medicine, University of Alabama at Birmingham, Birmingham, AL, United States

**Keywords:** neuroinflammation, mitochondria, neurological disorder, therapy, aging

## Abstract

The term “neurodegenerative diseases” (NDDs) refers to a range of aging-associated conditions, including Alzheimer’s disease, Parkinson’s disease, and amyotrophic lateral sclerosis. Unique clinical symptoms and underlying pathological mechanisms distinguish each of these illnesses. Although these conditions vary, they share chronic neuroinflammation as a defining characteristic. Protein aggregation and mitochondrial dysfunction are believed to play a role in initiating the neuroinflammatory response and, subsequently, the development and course of these illnesses. Apart from providing energy to the cells, mitochondria are involved in the immunoinflammatory response associated with neurological disorders such as Alzheimer’s disease, Parkinson’s disease, multiple sclerosis, and epilepsy. This involvement is attributed to controlling processes such as inflammasome activation and cell death. Under inflammatory conditions, the underlying regulatory mechanisms for these aging-associated disorders may include calcium homeostasis imbalance, mitochondrial oxidative stress, mitochondrial dynamics, and epigenetics. Various NDDs are linked to neuroinflammation and mitochondrial dysfunction. The linkages between these occurrences are becoming more apparent, but the etiology of these pathologic lesions is yet to be elucidated. This review examines the role of neuroinflammation and mitochondrial dysfunction in the growth and course of NDDs, emphasizing the possibility of identifying novel therapeutic targets to address aging-related neurodegenerative processes and retard the progression of these illnesses.

## 1 Introduction

The correlation between mitochondrial dysfunction and neuroinflammation plays a crucial role in the development and progression of various neurological disorders (de Oliveira et al., 2021). Neuroinflammation, which is thought to be a secondary response to injury, is now emerging as a major contributor to neurodegenerative diseases (NDDs), stroke, and mental conditions. This complex interrelationship creates a loop of bilateral etiology in which mitochondrial dysfunction promotes neuroinflammation, which worsens mitochondrial damage (Stuckey et al., 2021). This cycle sustains and intensifies neurodegenerative decline, resulting in increased neuronal activity loss and cognitive decline.

Mitochondria are the active parts of cells that generate energy via oxidative phosphorylation (OXPHOS), which uses the electron transport chain (ETC) to produce adenosine triphosphate (ATP) (Kuznetsov et al., 2022). Apart from generating energy, mitochondria participate in various cell-related activities such as calcium balance, reactive oxygen species (ROS) production, apoptosis, and signal transmission. Impairment in mitochondrial function leads to numerous cellular anomalies that eventually contribute to pathological conditions (Vercesi et al., 2018).

Neuroinflammation, on the contrary, is an immunological response within the central nervous system (CNS), causing the activation of glial cells (microglia and astrocytes) as well as the production of inflammatory molecules (Guzman-Martinez et al., 2019). Initially, this process was considered a secondary reaction to neural injury or infection but is now acknowledged as a key player in the onset of many neurological conditions, including Alzheimer’s disease (AD), Parkinson’s disease (PD), multiple sclerosis (MS), and stroke (Streit et al., 2004).

In addition to these inflammatory responses, damaged mitochondrial DNA (mtDNA) and damage-associated molecular patterns (DAMPs) are released by mitochondria into the extracellular space and the cytoplasm. DAMPs comprise proteins, DNA, and lipids. These chemicals trigger innate immunological reactions, exacerbating inflammation (Ji et al., 2023). These DAMPs may activate toll-like receptors and Nod-like receptors (NLRs), two types of pattern recognition receptors (PRRs) on microglia and astrocytes. This activation could cause chemokine and proinflammatory cytokine release. Activated microglia and astrocytes produce several inflammatory mediators (cytokines, chemokines, ROS, *etc.*), which modify the mitochondrial membrane potential, reduce ATP synthesis, and increase ROS generation in the mitochondria. These changes directly impact mitochondrial activity (Bernaus et al., 2020). Inflammatory cytokines, including TNF-α, IL-1β, and IL-6, impair mitochondrial function by modifying membrane potential, diminishing ATP synthesis, and elevating ROS generation. Berman et al. (2008) showed that cytokines induce mitochondrial fragmentation via DRP1 activation, resulting in depolarisation of the mitochondrial membrane potential (ΔΨm) and diminished efficiency of the electron transport chain. This disturbance reduces ATP production and undermines neuronal bioenergetics. Cytokine-induced dysfunction at complexes I and III simultaneously enhances electron leakage, resulting in elevated ROS levels and oxidative stress (Cassidy-Stone et al., 2008; Frank, 2006).

Moreover, chronic neuroinflammation disrupts calcium homeostasis, activates cell death pathways, and inhibits mitochondrial biogenesis, all of which contribute to mitochondrial breakdown and neuronal damage (Nakahira et al., 2015). Furthermore, problems such as neuronal loss, synaptic dysfunction, and cognitive decline may be caused by poor/compromised energy metabolism, oxidative stress, excitotoxicity, and inflammation-induced damage (Verma et al., 2023). These issues result in the spread of the disease throughout the CNS. This bilateral interplay (effect of neuroinflammation on mitochondrial dysfunction and *vice versa*) underscores the importance of addressing both mitochondrial dysfunction and neuroinflammation for effective therapeutic interventions in neurological disorders (Gao et al., 2023).

Another cause for neuroinflammation is endoplasmic reticulum (ER) dysfunction, which primarily results from stress responses triggered by protein mutation, viral infection, nutrient deprivation, hypoxic environment, *etc.* (Lin et al., 2008). As the ER is the main site for lipid production, calcium homeostasis, and protein folding, perturbation in its functions may compromise the health of cells, including neurons (Wake et al., 2012). The stress induced by protein misfolding in the ER turns off the unfolded protein response (UPR), which at first attempts to return to equilibrium; however, if left unchecked, it activates several pathways, including IRE1, PERK, and ATF6 (Pape et al., 2019; Wake et al., 2012; Tian et al., 2017; Read and Schröder, 2021). Activation of these pathways produces cytokines such as interleukin (IL)-6, tumor necrosis factor (TNF), and IL-1β as well as inflammatory signaling cascades such as nuclear factor-kappa B (NF-κB). This process initiates the inflammatory responses in affected cells, including B cells, myeloid cells, and hematopoietic stem cells (Rincon, 2012). Furthermore, ER stress disturbs calcium homeostasis and increases oxidative stress by producing ROS, which intensifies inflammatory reactions and glial activation (Mondelli et al., 2017; Khandaker et al., 2017). Prolonged ER dysfunction encourages proinflammatory phenotypes in microglia and astrocytes, releasing cytokines, chemokines, and ROS that damage synapses and neurons (Veeresh et al., 2019). Dysfunctional ER also affects mitochondrial activity as the two organelles are connected via mitochondria-associated membranes (Missiroli et al., 2018; Yao et al., 2023). ER stress disrupts mitochondrial function, increases ROS generation, and exacerbates inflammation in a vicious cycle. In addition, pathogens that use the ER for replication can cause ER stress, fueling inflammatory signals in the CNS (Zhang et al., 2023). However, discussing the detailed mechanism of stress-mediated neuroinflammation is outside the scope of this review.

Therapeutic techniques that protect mitochondrial activity and alleviate neuroinflammation offer possibilities for effectively managing neurological diseases (Kumar and Singh, 2015). Therefore, substances that target mitochondria (such as coenzyme Q10), pharmaceuticals that decrease inflammation [nonsteroidal anti-inflammatory drugs (NSAIDs)], and compounds that alter the immune system may help treat neuroinflammation and overcome the adverse effects on mitochondrial function (Kip and Parr-Brownlie, 2022).

Understanding the intricate relationship between the development of NDDs, neuroinflammation, and mitochondrial failure is critical for identifying novel therapeutic targets and developing effective treatments to prevent or reduce disease development. Interventions that address both mitochondrial failure and neuroinflammation hold promising potential for alleviating neuronal damage and enhancing the prospects of patients suffering from these complex pathologies. This review examines the multifaceted connections between defective mitochondria and neuroinflammation, revealing their interconnected roles in the etiology of neurological diseases. By investigating the molecular mechanisms underlying this link and its implications for neuronal health, we hope to gain insights into future treatment strategies and the development of novel therapies. This understanding may help retard or even reverse disease development, and help in the management of these disease.

## 2 Mitochondria and their role in neuronal function

**FIGURE 1:**
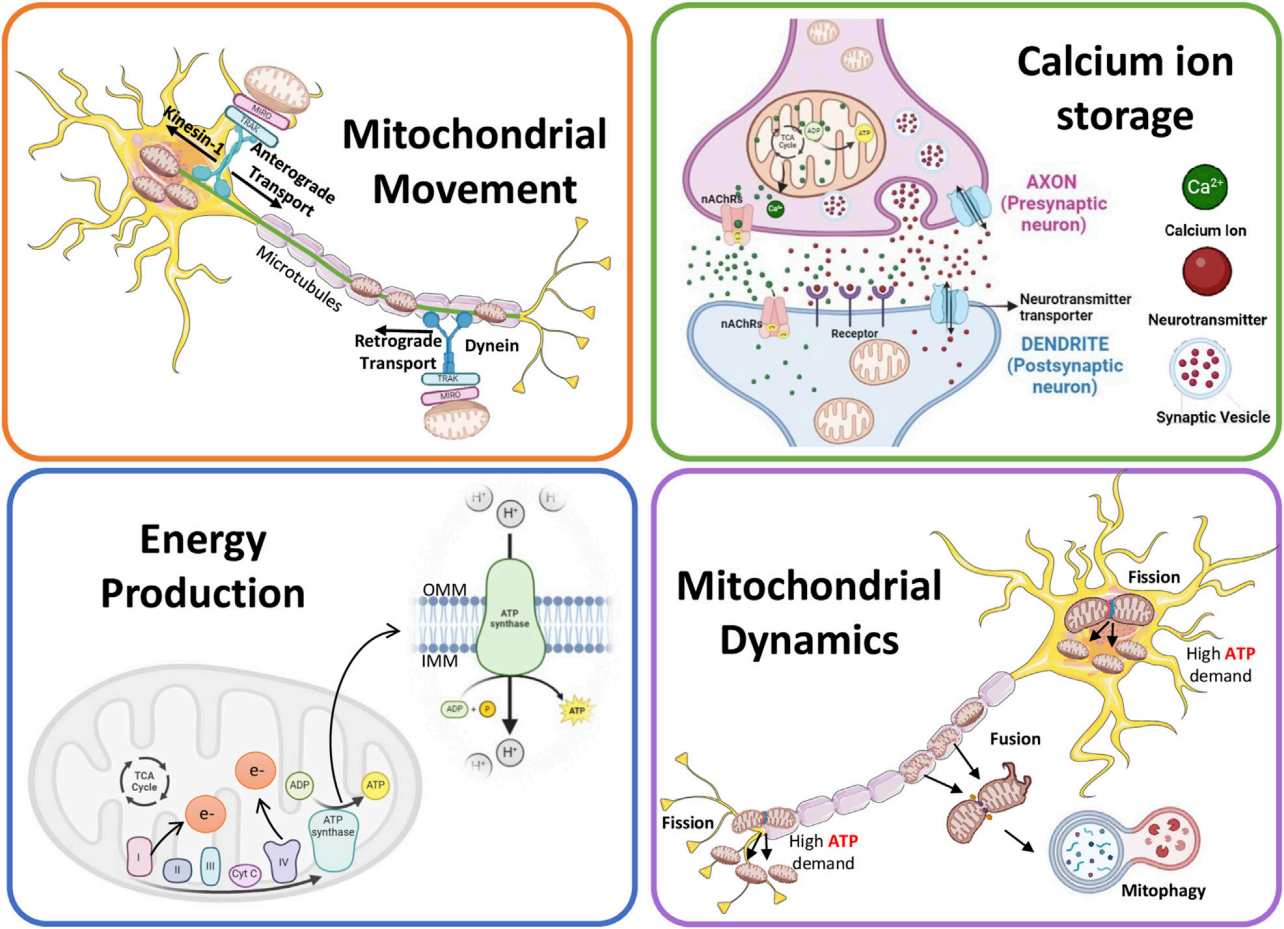
Schematic showing the role of mitochondria in neuronal functions.

Neurons require high energy due to their complex morphology and high polarizability (Marshall et al., 2023; Seager et al., 2020). The mitochondria help overcome physiological disturbances that could change the structure and function of neurons by producing ATP, and thus preserving neuroplasticity (Bramham, 2008; Shah et al., 2010). Examples include axon/dendrite growth, synapse formation, and neurogenesis. The process of neuronal plasticity involves diverse molecular mechanisms such as neurotransmission, membrane trafficking, protein synthesis, and gene transcription (Bramham, 2008; Cheng et al., 2010; Shepherd and Huganir, 2007; Tai and Schuman, 2008). To support the plastic nature of neurons, differences exist in mitochondrial shape between the dendritic and axonal compartments. Mitochondria in the dendrite are long and occupy a large neurite volume, whereas those in the axon are small and scanty in distribution (Chang et al., 2006; Lewis et al., 2018). Moreover, the axonal and dendritic mitochondria exhibit varied responses to neuronal activity, showing differences in metabolism and movement (Chang et al., 2006), with axonal mitochondria showing a higher dynamic activity than dendritic mitochondria (Rangaraju et al., 2019a). The role of mitochondria in neuronal functions is summarized in Figure 1.

### 2.1 Mitochondrial movement and neuronal functions

Mitochondrial anchoring and transport are essential to compensate for the altered energy demands at different ends of neuronal cells. Cells carefully control their mitochondrial movement to balance energy requirements and prevent death (Hollenbeck and Saxton, 2005). Because of their extremely extended architecture and specific functions, neurons are especially prone to interruptions in the dispersion and mobility of mitochondria. The control of mitochondrial mobility is crucial for both neuronal survival and degeneration (Lovas and Wang, 2013).

Two types of mitochondrial movements occur in neurons: anterograde and retrograde (Mandal and Drerup, 2019). Both these mitochondrial movements, throughout the neurons, are supported by cytoplasmic dynein motors for retrograde and kinesin-1 for anterograde movement (Hollenbeck and Saxton, 2005; Mandal et al., 2021; Pilling et al., 2006; Schnapp and Reese, 1989; Tanaka et al., 1998). Anterograde transport helps axon extension and regeneration for proper signal transmission by moving healthy mitochondria toward axon terminals and away from the cell body (Han et al., 2016; Morris and Hollenbeck, 1993; Ruthel and Hollenbeck, 2003; Spillane et al., 2013; Zhou et al., 2016). The injured mitochondria, on the other hand, migrate away from the axon terminals because retrograde mitochondrial transport is mostly in charge of removing and labeling damaged organelles and starting their breakdown within the cell body (Cai et al., 2012; Mandal et al., 2021; Frederick and Shaw, 2007; Miller and Sheetz, 2004). Trak1/2 (Guo et al., 2005; Stowers et al., 2002) and Rho GTPase 1 (Rhot1) are the two main adapter proteins implicated in anterograde migration. While Trak2 predominantly binds to dynein, Trak1 also attaches to kinesin-1. Nevertheless, insufficient proof exists to draw a firm connection between Trak2 activity and retrograde transit (van Spronsen et al., 2013). Actr10 is a different protein that helps mitochondria travel along the cytoskeleton in healthy neurons, ensuring these organelles are distributed and operate correctly. A critical link between dynein and mitochondria was established when deleting the *Actr10* gene from zebrafish neurons impaired only the retrograde but not anterograde mitochondrial transport or the transport of other cargo (Drerup et al., 2017). Their movement mechanism must be studied to comprehend the function of mitochondria in neurons.

Mitochondrial movement is also responsible for removing aging and damaged mitochondria from the distal ends of neurons and maintaining neuron health (Palikaras et al., 2018). Mitochondrial mobility promotes mitophagy by guaranteeing that damaged mitochondria are carried retrogradely toward the cell body, where the autophagic machinery is more prevalent. Once within the cell body, damaged mitochondria are identified by mitophagy receptors and are removed selectively (Sheng, 2014).

### 2.2 Ca^2+^ storage and neuronal functions

The optimal calcium ion (Ca^2+^) level is key to neuronal health. Several neuronal functions, such as the release of neurotransmitters, synaptic plasticity, and signal transmission, are highly dependent on Ca^2+^ homeostasis (Jedrzejewska-Szmek et al., 2023). As important secondary messengers in various processes necessary for efficient communication within the nervous system, Ca^2+^ is pivotal for transmitting signals in neurons (Brini et al., 2014). An action potential triggers the opening of voltage-gated Ca^2+^ channels that reach the axon terminal of a neuron, letting Ca^2+^ enter the cell (Iosub et al., 2015; Werth and Thayer, 1994). Neurotransmitters are released into the synaptic cleft when synaptic vesicles fuse with the presynaptic membrane due to Ca^2+^ inflow. The signal can then continue to be transmitted once these neurotransmitters attach themselves to postsynaptic neuron receptors (Littleton et al., 1999). Long-term potentiation and depression, two synaptic plasticity mechanisms vital for learning and memory, are supported by this process and rely heavily on Ca^2+^ signaling. Depending on the activity, Ca^2+^ triggers different signaling pathways and kinases that alter the structure and responsiveness of synapses by either strengthening or weakening synaptic connections (Abraham et al., 2019). Ca^2+^ also activates various transcription factors (such as cAMP response element-binding protein, nuclear factor of activated T-cells, and myocyte enhancer factor-2), thus regulating the expression of crucial genes that influence differentiation, growth, and neuronal response to external stimuli (Bagur and Hajnoczky, 2017). Moreover, the optimal level of Ca^2+^ ensures appropriate neural circuit creation during neuronal development as the Ca^2+^ gradients direct growth cones and control neurite outgrowth (Park and Poo, 2013).

Synaptic mitochondria mediate the process of buffering and transfer of intracellular Ca^2+^ levels, facilitating neurotransmission (Billups and Forsythe, 2002; Kann and Kovacs, 2007; Kim et al., 2011; Levy et al., 2003; Rizzuto et al., 2012). By rapidly absorbing and releasing Ca^2+^ via the Na^+^/Ca^2+^ exchanger and mitochondrial calcium uniporter (MCU), respectively (as explained later in the section), these organelles can maintain Ca^2+^ homeostasis (Shoshan-Barmatz and De, 2017). Mitochondria mitigate the cytotoxicity linked to increased Ca^2+^ levels by sequestering surplus Ca^2+^ during neural activation and regulating the spatiotemporal dynamics of Ca^2+^ signaling, ensuring proper neuronal survival and function (Giorgi et al., 2018a).

Voltage-gated Ca^2+^ channels at the presynaptic terminal activate during neuronal depolarisation, facilitating Ca^2+^ influx and initiating neurotransmitter release (Sudhof, 2012). The mitochondrial membrane potential, produced by the electron transport chain, enables Ca^2+^ influx into the mitochondrial matrix (Kann and Kovacs, 2007; Rizzuto et al., 2012). Baseline cytosolic and mitochondrial Ca^2+^ concentrations are approximately 100 nM, but increase during Na^+^ channel-mediated depolarisation. Dendritic mitochondria mitigate this rise and assist in reinstating the resting potential (Billups and Forsythe, 2002; Burke et al., 2001). Calcium ions (Ca^2+^) ingress into mitochondria through voltage-dependent anion channels and the mitochondrial calcium uniporter (MCU), modulating synaptic efficacy and neurotransmitter secretion (Giorgi et al., 2018b; Kim et al., 2011). Mitochondrial depolarisation influences mtDNA and synaptic vesicle dynamics (Kim et al., 2011). Fusion improves Ca^2+^ uptake efficiency by altering mitochondrial structure and membrane potential (Kowaltowski et al., 2019; Trevisan et al., 2018). Dendritic mitochondria in the postsynaptic neuron undergo fission to modulate Ca^2+^ uptake and sustain signalling efficacy (Divakaruni et al., 2018; Han et al., 2008; Rangaraju et al., 2019a). Mitochondrial Ca^2+^ furthermore activates tricarboxylic acid cycle enzymes, enhancing ATP synthesis to fulfil neural energy requirements (Denton et al., 1978; Jouaville et al., 1999).

### 2.3 Energy production and neuronal function

Neurons are particularly demanding because of their intricate signal transmission and information processing functions. The balance in mitochondrial metabolism is integral for sustaining neurotransmission, preserving ion gradients throughout the cell membrane, facilitating synaptic formation and plasticity, and releasing synaptic vesicles (Cai and Tammineni, 2017; Hall et al., 2012). Mitochondrial ATP production plays a pivotal role in fulfilling this high energy demand, ensuring appropriate neuronal functioning (Rossi and Pekkurnaz, 2019). Numerous studies have elucidated the critical relationship between mitochondrial ATP production and neuronal activities. For instance, research has highlighted the significance of OXPHOS in generating ATP within neuronal mitochondria under high energy demands rather than depending on glycolysis.

Deteriorated mitochondrial ATP production can result in energy deficits, compromising neuronal viability and functionality (Clemente-Suarez et al., 2023). Regardless of their primary causation, disturbances in mitochondrial functions contribute to neuronal demise and are implicated in various neurological conditions, such as amyotrophic lateral sclerosis (ALS), PD, AD, Huntington’s disease (HD), spinocerebellar ataxia and peripheral neuropathies such as Charcot–Marie–Tooth disease (Pass et al., 2021). Observable signs of mitochondrial dysfunction include decreased respiratory chain complex activity, which always affects ATP synthesis (Zhao et al., 2022). Furthermore, recent research has focused on the association between mitochondrial function and aging, reporting that mitochondria become less efficient with age, leading to decreased neuronal function. This mitochondrial performance reduction has been further linked to age-related cognitive decline and NDDs (Chistiakov et al., 2014).

### 2.4 Mitochondrial dynamics and neuronal functions

Mitochondria undergo constant fission and fusion, along with mitophagy, to maintain their quality and function. These dynamic processes are essential for neuronal health (Cheng et al., 2010; Detmer and Chan, 2007). The intricate interaction of molecular machinery closely controls these processes. Mitofusins (MFN1 and MFN2) mediate fusion of outer mitochondrial membrane (OMM) by forming homo- and heterodimers (Cipolat et al., 2004). OPA1 facilitates inner mitochondrial membrane (IMM) fusion (MacVicar and Langer, 2016). Fission is driven by DRP1, a GTPase that oligomerizes at the OMM to constrict and divide mitochondria, with support from mitochondrial fission protein 1 FIS1 (Fonseca et al., 2019; Otera et al., 2010).

Fission enables mitochondrial transport to energy-demanding regions like axons and synapses, ensuring ATP availability for neural signaling. It also isolates damaged segments for removal via mitophagy (Trevisan et al., 2018). On the other hand, the process of fusion supports mitochondrial integrity by forming networks that enhance bioenergetics, calcium buffering, and synaptic function (Berman et al., 2008; Westermann, 2010).

Apart from fission and fusion, mitophagy is also an essential cellular quality control process. It involves the breakdown of defective and unnecessary mitochondria via macroautophagy. This safeguarding mechanism ensures the optimal functioning of the mitochondrial network, enabling it to adapt to the constantly changing physiological requirements (Kanki et al., 2010; Sarraf et al., 2013; Palikaras et al., 2018; Yamashita et al., 2016). Proteins such as OPA-1, DRP1, and MFN2 not only initiate mitophagy but also influence the production of autophagosomes and regulate mitochondrial activity. In addition, the start of mitophagy requires PTEN-induced putative kinase 1 (PINK1) and E3-ubiquitin ligase protein (parkin). To ubiquitinate target proteins, these proteins accumulate on the OMM (Lazarou et al., 2013; Wibawa et al., 2015). In a well-studied mitophagy model, a disturbed membrane potential keeps PINK1 from entering mitochondria and stabilizes it on the outer membrane (Narendra et al., 2010). Subsequently, PINK1 phosphorylates ubiquitin moieties on various protein substrates, including parkin, initiating its recruitment and activation in the proximity of the OMM (Ng et al., 2014). Activated parkin ubiquitinates OMM protein, enabling the recognition of autophagic adapters on their journey toward mitochondrial degradation in the lysosomes.

The well-established correlation between mutation in parkin and PINK1 and the prevalence of autosomal recessive cases of PD highlights the importance of this complex regulatory mechanism (de Oliveira et al., 2021). Mitophagy and autophagy are crucial for promoting longevity, and both these processes tend to decline with advancing age (Palikaras et al., 2017). Inhibiting mitophagy has been linked to a reduced lifespan, whereas fostering autophagy has been associated with an extended lifespan (Kissova et al., 2004; Melendez et al., 2003). While the mechanisms underlying these relationships are not completely understood, efficient mitophagy has been hypothesized to mitigate mitochondria-associated oxidative stress and lessen the number of damaged mitochondria with mtDNA mutations. The neurological system is particularly prone to mitochondrial malfunction because of its increased energy requirements. Impaired mitophagy is implicated in neuropathologies observed in certain familial variations of HD, AD, and PD, making it a potential therapeutic target (Trigo et al., 2022).

## 3 Role of neuroinflammation in neurological diseases

Neuroinflammation is an immunological reaction within the central nervous system, predominantly characterised by the activation of microglia and astrocytes (Kwon and Koh, 2020; DiSabato et al., 2016). It may be induced by infections, trauma, autoimmune illnesses (metabolic abnormalities, and environmental pollutants (Giri et al., 2024). Other contributing factors include stress, ageing, mitochondrial malfunction, and disturbance of the gut–brain axis (Adamu et al., 2024). Chronic neuroinflammation results in synaptic impairment, diminished plasticity, and neuronal degeneration, which are associated with disorders such as schizophrenia, depression, and neurodegeneration (Monji and Mizoguchi, 2021). Understanding these pathways is crucial for the development of therapeutics, including anti-inflammatory drugs, immunomodulators, and antioxidants (Moyse et al., 2022).

**FIGURE 2:**
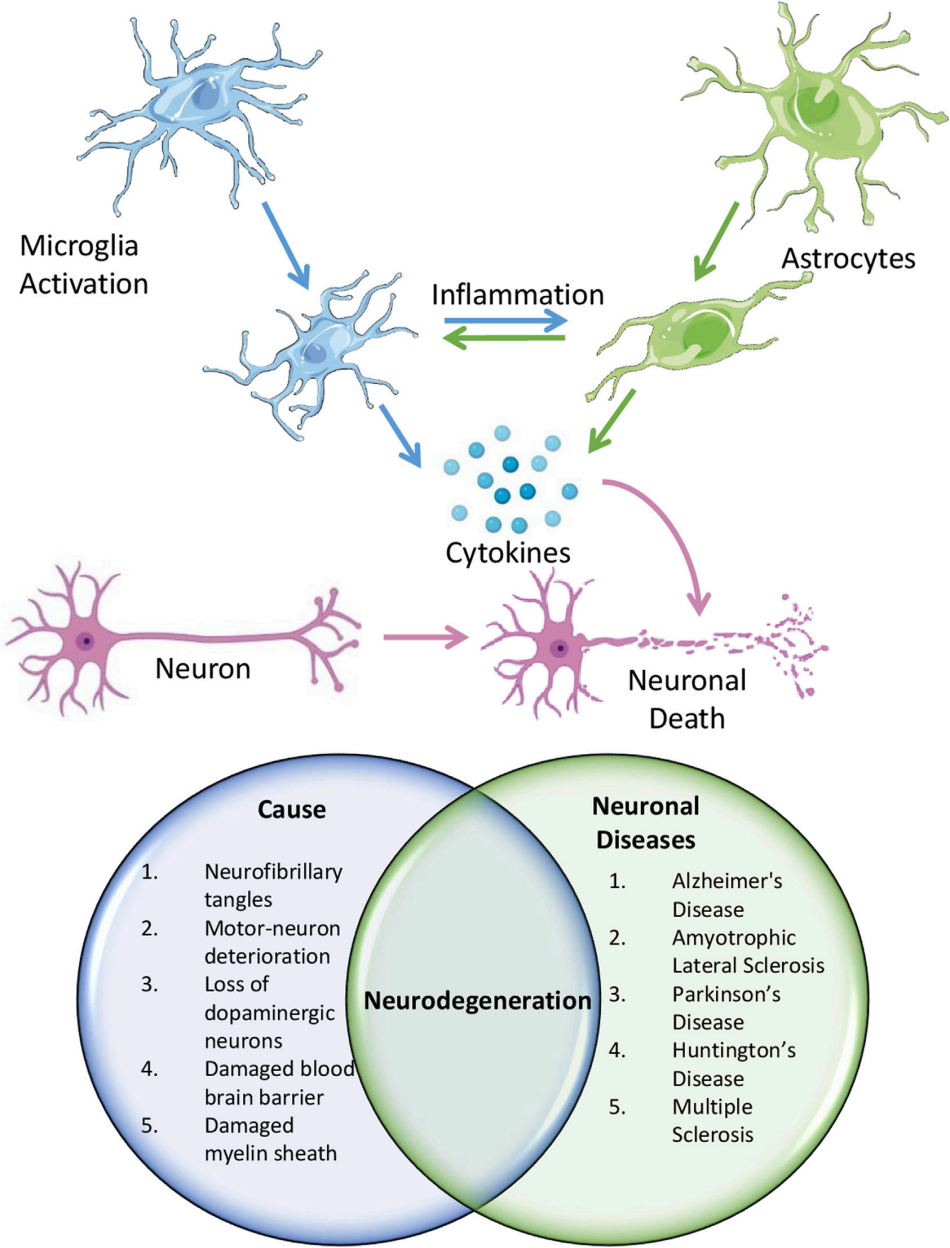
Schematic showing the role of neuroinflammation in causing different neurological diseases.

NDDs encompass conditions that impact both the CNS (spinal cord and brain) and the peripheral nervous system (nerves, ganglia, sensory nerves, motor nerves, and autonomic nervous system) in humans (Borsook, 2012). These include progressive neurological diseases (AD, PD, and HD), developmental brain disorders [Schizophrenia, autism spectrum disorders (ASD), and certain forms of epilepsy], cerebrovascular issues (stroke), and neuroinfectious diseases (meningitis and encephalitis) (Clemente-Suarez et al., 2023). Persistent or severe neuroinflammation, particularly in the CNS, may lead to the emergence and advancement of several neurological illnesses (Figure 2).

### 3.1 Alzheimer’s disease

Neuroinflammation is significantly associated with the pathogenic aspect of Alzheimer’s and is characterized by a complex interplay of cellular and molecular mechanisms that contribute to progressive neurodegeneration. Glial cell stimulation, immune cell translocation from the peripheral system, amyloid beta (Aβ) plaque accumulation, and neurofibrillary tangles (NFTs) are components of the neuroinflammatory response in AD (Heneka et al., 2015). Cerebral inflammation in Alzheimer’s disease is distinguished by the stimulation of microglial cells and astrocyte cells at the cellular scale. When activated, these supportive brain cells secrete various proinflammatory substances, including cytokines (such as interleukin 1β and 6 and TNF), chemokines, ROS, and other inflammatory mediators that trigger an immune response. This inflammatory milieu exacerbates neurotoxicity, leading to excessive neural cell death and fueling the neuroinflammatory cascade. The invasion of immune cells in the outer regions, including lymphocytes and monocytes, into the brain parenchyma also occurs in AD, amplifying the inflammatory response. These invading cells secrete extra-inflammatory substances and continue the cycle of neuroinflammation and neurodegeneration (Kaur et al., 2019). The major contributor to neuroinflammation in AD is the accumulation of Aβ plaques and NFTs, which induces neurotoxicity, leading to excessive neural cell death via apoptosis, autophagy, and necroptosis (Onyango et al., 2021). This process establishes a positive feedback loop in which elevated neuroinflammation results in neurodegeneration. At the molecular level, neuroinflammation in AD disrupts transcriptional mechanisms, affecting the expression of genes encoding proinflammatory cytokines such as IL-1β, 6, and TNF. NF-κB controls microglial activation to regulate the process of neuroinflammation. In addition, activating the inflammasome NLRP3 in microglia promotes IL-1β production, leading to persistent inflammatory reactions in AD (Li et al., 2022). The buildup of Aβ in mitochondria disrupts mitochondrial function and increases the production of ROS that activate inflammatory signaling pathways, exacerbating neuroinflammation. Excessive accumulation of Aβ and tau impairs autophagic function, hindering protein clearance and augmenting neuroinflammation and neuronal degeneration (de la Cueva et al., 2022).

### 3.2 Parkinson’s disease

In Parkinson’s, an intricate network of cellular and molecular mechanisms collaborates to degenerate nerve cells that produce dopamine within the substantia nigra pars compacta part of the midbrain. During this inflammatory reaction, reactive glial cells release numerous inflammation-promoting cytokines, chemokines, ROS, and other inflammatory mediators, creating a toxic environment that exacerbates nerve cell deterioration. The dysregulation of cytokine levels is a key aspect of neuroinflammation in PD (Di Lazzaro et al., 2023). Neurodegeneration is accelerated in the brain of patients with PD owing to increased amounts of inflammation-promoting signaling molecules, such as IFN-γ, IL-1β, 6, and TNF (Isik et al., 2023). Moreover, anti-inflammatory signaling molecules, such as IL-10 and transforming growth factor beta (TGF-β), are improperly regulated, further disturbing the delicate equilibrium of inflammatory mediators (Belarbi et al., 2020). In PD, glial cells are activated, and immune-specific cells such as T lymphocytes, monocytic cells, and macrophages from the external environment infiltrate the brain parenchyma. This infiltration increases the potency of the inflammatory response. These infiltrating cells can release additional inflammatory mediators and exacerbate the neuroinflammatory cascade (Munoz-Delgado et al., 2023).

At the molecular level, genetic factors are crucial in the development of PD and the initiation of neuroinflammatory processes. Genetic alterations in several proteins have been indicated in the accumulation of misfolded PD-associated proteins. These changes encompass genetic variations in the genes encoding α-synuclein (*SNCA*), glucocerebrosidase (*GBA*), vacuolar protein sorting 35 (*VPS35*), and leucine-rich repeat kinase 2 (*LRRK2*). These modifications activate inflammatory signaling pathways, triggering glial cell activation (Yao et al., 2021). Neuroinflammation in PD also involves the dysregulation of transcriptional processes, affecting the expression of inflammatory genes. NF-κB controls the production of different cytokines that promote inflammation and has a vital function in activating microglia and, thus, the inflammation process (Tiwari and Pal, 2017).

Microglia, the primary agents in brain inflammation (Woodburn et al., 2021), can assume either an M1 or an M2 macrophage state. Microglial transformation to the M1 phenotype from the M2 state contributes to neuroinflammation and neurodegeneration in PD. In addition, NLRP3 inflammasome activation in microglia releases pro-inflammatory cytokines such as IL-1β, intensifying neuroinflammation in PD. Mitochondrial dysfunction and increased ROS synthesis, which are hallmarks of PD pathology, can also activate inflammation-associated molecular pathways. This interaction among inflammation, oxidative damage, and mitochondrial impairment establishes a self-sustaining cycle that propels the ongoing degeneration of dopamine-producing neurons in PD (Pizarro-Galleguillos et al., 2023).

### 3.3 Huntington’s disease

The primary factor responsible for immune-mediated inflammation and dysregulated molecular pathways observed in HD is the accommodation of mHtt (mutant huntingtin protein) containing an elongated polyglutamine repeat. mHtt initiates a self-perpetuating cycle of neuroinflammation and neurodegeneration via multiple mechanisms. Initially, the misfolding and aggregation of mHtt activate the NF-κB signaling pathway without any intermediaries or NLRP3 inflammasome (Saba et al., 2022). These aggregates also impair protein homeostasis by interfering with the ubiquitin–proteasome system and autophagic processes, accumulating improperly folded proteins. This buildup activates UPR and induces stress in the ER, exacerbating inflammatory reactions. Furthermore, mHtt associates with mitochondria, leading to mitochondrial impairment and elevated ROS generation, triggering inflammatory signaling cascades and promoting the release of immune-activating cytokines. The presence of mHtt aggregates and the associated stress further activate microglia along with astrocytes, producing inflammatory mediators (Saba et al., 2022). Transcriptional dysregulation is another key aspect of HD pathogenesis. mHtt interferes with transcriptional regulators such as CREB-binding protein and histone deacetylases (HDACs), altering gene expression, including that of inflammatory genes. Furthermore, mHtt can induce epigenetic changes, such as histone modifications and DNA methylation, affecting the expression of proinflammatory and anti-inflammatory genes. Moreover, mHtt disrupts the function of transcriptional regulators implicated in immune responses, including NF-κB, AP-1, and STAT3, modifying the expression of their downstream genes (Pogoda et al., 2021). Impaired clearance mechanisms exacerbate this cycle as inflammation and autophagy dysfunction impair the clearance of mHtt aggregates, enhancing their accumulation (Pircs et al., 2022). Comprehending the intricate neuroinflammation pathway and the underlying molecular biology in HD is crucial to developing precise therapeutic strategies that can break the vicious cycle and potentially slow the progression of this devastating neurodegenerative disease.

### 3.4 Multiple sclerosis

MS is a condition in which the body’s immune system erroneously targets and damages the protective myelin sheath in the CNS. Autoreactive myeloid cells and lymphocytes invade the CNS, triggering an inflammatory response that drives the pathogenesis of MS (Papiri et al., 2023). On the cellular scale, brain inflammation in MS is marked by stimulating microglial cells, glial cells, and oligodendrocytes within the CNS (Amoriello et al., 2024). These reactive supportive cells secrete diverse inflammatory signaling molecules, fueling the inflammatory cascade. Dysregulation of cytokine levels, particularly those of TNF, IL-17, and granulocyte–macrophage colony-stimulating factor (GM-CSF), contributes to the pathogenesis of MS (Amoriello et al., 2024). The inflammatory environment leads to synaptopathy, a process characterized by synaptic loss, reduced synaptic protein levels, and altered neurotransmission in MS (Mandolesi et al., 2014; Nisticò et al., 2017; Depino et al., 2003). Cytokines such as TNF modulate glutamatergic neurotransmission by altering the expression and functional properties of AMPA receptors, contributing to synaptic dysfunction. This dysregulation of neurotransmission precedes the symptomatic phase in animal models of MS (Bruno et al., 2020). Activated microglia near axon terminals cause local calcium overload and spine removal, exacerbating neuronal damage. Cytokines such as GM-CSF stimulate the activation and growth of microglial cells, resulting in altered neural network patterns and excitability. Moreover, various cytokines, such as IL-17 and GM-CSF, can interact and worsen neuronal alterations during disease exacerbations, highlighting the complexity of the neuroinflammatory processes in MS (Amoriello et al., 2024). At the molecular level, neuroinflammation in MS involves epigenetic changes and dysregulation of transcriptional processes, affecting inflammatory gene expression (Huynh and Casaccia, 2013). These epigenetic modifications and transcriptional dysregulation perpetuate the inflammatory cascade and neurodegeneration. The neuroinflammatory processes and molecular mechanisms in MS culminate in synaptopathy, neurodegeneration, and the development of neurological deficits in patients (Ellwardt and Zipp, 2014).

### 3.5 Amyotrophic lateral sclerosis

Neuroinflammation in the CNS is a prominent feature of ALS and is observed in both experimental setups and individuals diagnosed with this disorder. The invading immune cells, as well as resident microglial cells and astrocytes, activate the immune reaction and interact with multiple cellular components. Microglia play a vital role in the process of neuroinflammation in ALS. These cells can assume either a harmful M1 or a beneficial M2 phenotype. After the initiation of ALS in mouse models, the cellular transition of microglia from the M2 to the M1 state leads to neural inflammation and disease progression (Geloso et al., 2017). In addition, activating the NLRP3 inflammasome in microglia is a prominent factor that promotes neuroinflammation in ALS. Astrocytes, a specific type of brain cells that provide support, are also involved in the inflammatory process in ALS. These cells secrete a range of signaling proteins, including proinflammatory and anti-inflammatory mediators. Proinflammatory molecules such as IL-1, IL-6, and TNF and anti-inflammatory mediators such as prostaglandin E2 and TGF-β are mainly released (Peggion et al., 2024). Astrocytes generated from fibroblasts of ALS patients, via trans-differentiation method, exhibited toxicity toward motor neurons cocultured with them, possibly due to compromised bioenergetic support or impaired signaling of pro-nerve growth factors (Gomes et al., 2022). Infiltration of lymphocytes and macrophages into the CNS is also noted in ALS, amplifying the inflammatory response. The NF-κB protein is a pivotal regulator of inflammation in ALS, controlling microglial activation and the occurrence of neuroinflammation (Geloso et al., 2017). At the molecular level, neuroinflammation in ALS involves the dysregulation of transcriptional processes, altering the gene expression of several inflammatory mediators. ER stress and RNA metabolism disruption have also been implicated in the development of ALS, potentially contributing to neuroinflammation. This complex interplay of cellular players and molecular mechanisms drives the neuroinflammatory processes in ALS, ultimately resulting in motor neuron dysfunction and death.

### 3.6 Other neurological disorders

Neuroinflammation is pivotal in the pathophysiology of schizophrenia, epilepsy, and autism spectrum disorders (ASD) by impairing neuronal connection, neurotransmission, and cerebral development. In schizophrenia, increased cytokines such as IL-6 and TNF-α disrupt dopamine and glutamate pathways, exacerbating symptoms, while hyperactive microglia induce excessive synaptic pruning and impair the blood–brain barrier, leading to additional neurotoxicity (Mosquera et al., 2024; Ermakov et al., 2023; Ayilara and Owoyele, 2023). Early-life inflammatory exposures, including maternal infections, elevate the risk of schizophrenia by disrupting brain development, as evidenced by heightened microglial activity and elevated cytokine levels (Buckley, 2019). These results have initiated trials of anti-inflammatory treatments, encompassing NSAIDs, monoclonal antibodies, and lifestyle modifications (Sommer et al., 2011; Essali et al., 2019; Gurusamy et al., 2024). In epilepsy, proinflammatory cytokines such as IL-1β and TNF-α increase neuronal excitability and diminish GABAergic inhibition, facilitating seizures via blood-brain barrier disruption, neuronal degeneration, and dysfunctional synaptic networks (Pracucci et al., 2021; Sanz et al., 2024; Li et al., 2023). Seizures exacerbate inflammation, establishing a detrimental cycle, with medications aimed at IL-1β and TNF-α pathways currently under examination (Ravizza et al., 2024; Panina et al., 2023; Mukhtar, 2024). In ASD, neuroinflammation impairs neuronal differentiation and synaptic integrity through cytokines generated by activated glial cells (Xiong et al., 2023; Usui et al., 2023). Maternal immune activation during gestation increases the likelihood of autism spectrum disorder by modifying foetal neural circuit development, whereas postnatal inflammation impacts brain areas such as the prefrontal cortex and amygdala, leading to fundamental behavioural abnormalities (Yin et al., 2023; Tan et al., 2024; Hughes et al., 2022). In genetic mouse models of ASD, cerebellar and systemic inflammation, often accompanied by oxidative stress, contribute to behavioural and motor deficits. N-acetylcysteine (NAC) treatment improved these impairments in Cntnap2 and Shank3b mutant mice, highlighting the interplay between neuroimmune dysfunction, oxidative stress, and aging in ASD pathophysiology (Pangrazzi et al., 2025; Pangrazzi et al., 2024; Cerilli et al., 2024).

## 4 Mitochondrial dysfunction and neuroinflammation: a complex interplay

**FIGURE 3:**
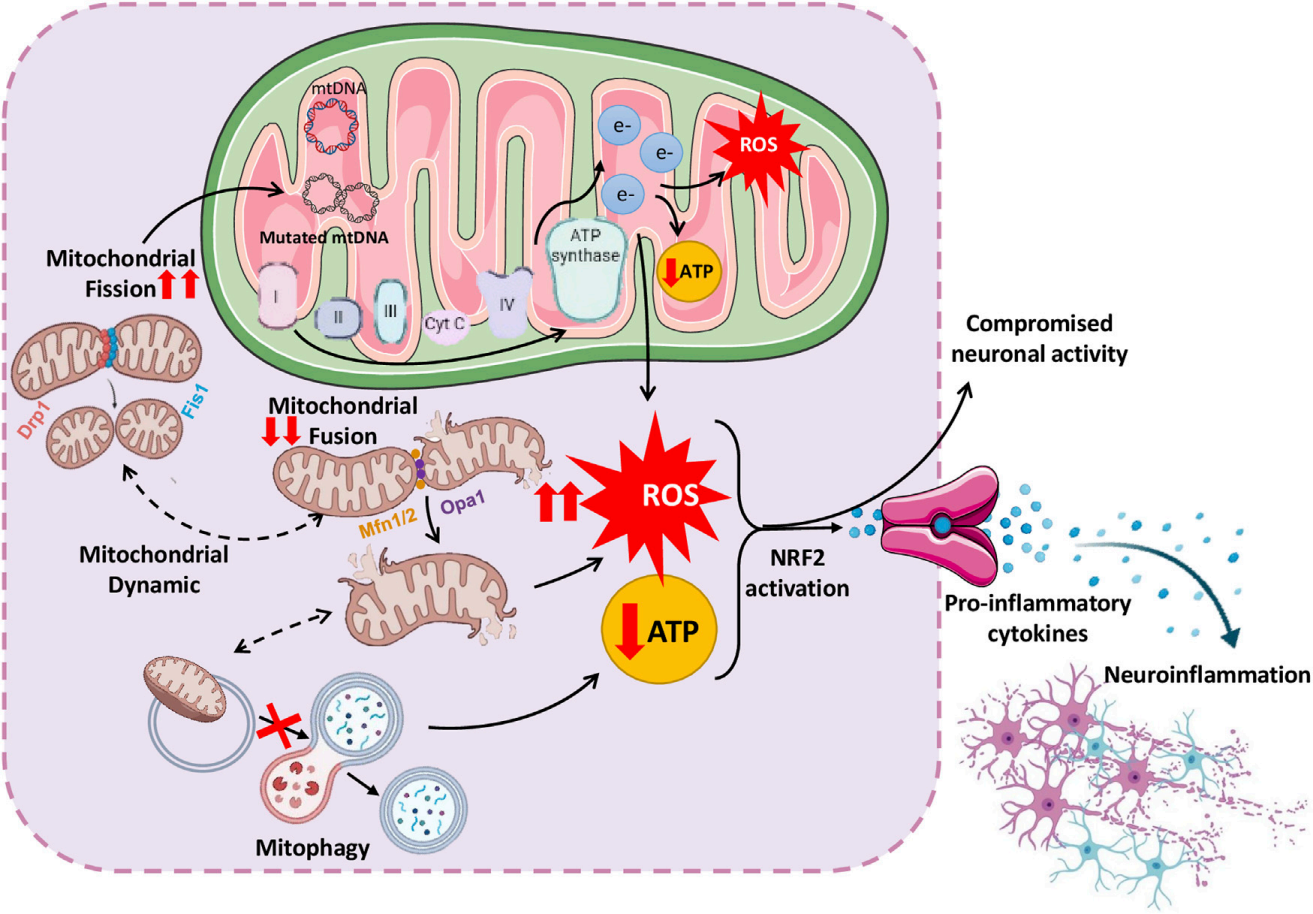
Schematic representation of complex interplay between mitochondrial dysfunction and neuroinflammation, leading to various neurological disorders.

NDDs are characterized by progressive brain cell loss and are linked to impairment of energy-producing organelles as well as immune-mediated inflammation in the CNS. Mitochondrial dysfunction initiates neuroinflammation by releasing mitochondrial damage-associated molecular patterns (DAMPs), which include mitochondrial DNA (mtDNA), proteins, and lipids, acting as danger signals, alerting the immune system to cellular malfunction. Mitochondrial dysfunction activates the redox-sensitive factor NF-κB pathway and also induces NLRP3 inflammasome activation. NLRP3 inflammasome/NF-kB pathways work together to activate inflammatory cytokines such as IL-1β and TNF. These inflammatory molecules activate microglia, causing ongoing inflammation in the brain and worsening neuronal damage (Wang et al., 2019). These inflammatory mediators exacerbate mitochondrial dysfunction, resulting in a self-perpetuating cycle of impaired function (Lin et al., 2022). Thus, the interplay between altered mitochondrial function and inflammation synergistically induces nerve cell death via excitotoxicity, oxidative stress, and defective energy metabolism, accelerating neurodegenerative disease pathology (de Oliveira et al., 2021; Kann and Kovacs, 2007) (Figure 3).

Defects in mitochondria also affect their dynamics, disturbing the balance between the fusion, fission and mitophagy processes. The disturbance in the balance between fission, fusion and mitophagy causes excessive ROS generation, energy insufficiency, and neuronal strain (de Oliveira et al., 2021). The following sections discuss important studies supporting mitochondrial dysfunction and their contribution to neuroinflammation and *vice versa*. Insights from studies on these interactions may lead to innovative therapeutic strategies that target inflammation and mitochondrial dysfunction, potentially retarding or stopping disease progression (Peggion et al., 2024).

### 4.1 Mitochondrial dysfunction and neuroinflammation

#### 4.1.1 Mutations in mitochondrial DNA

The complex correlation between mitochondrial functioning and nervous system wellbeing, particularly the impact of mutations in mtDNA on conditions such as AD, PD, and ALS, has recently received considerable attention. Neurons are highly susceptible to oxidative stress, which can result in somatic mutations in their mtDNA. Hence, acquired mutations in mtDNA can accumulate primarily in the brain and contribute to aging and neurodegenerative diseases. Emerging evidence suggests that mutations in mtDNA disrupt cellular energy metabolism and contribute to neuroinflammatory processes (Hahn and Zuryn, 2019; Kraytsberg et al., 2006). A proposed pathway involves ROS generation within dysfunctional mitochondria. ROS, being highly reactive molecules, can inflict cell and tissue injury and provoke an inflammatory reaction in the CNS. In addition, mutations in mtDNA can decrease ATP synthesis, causing stress in brain cells and releasing proinflammatory signals, which trigger an immune response in the CNS. Alongside human studies, animal research has strengthened the link between mtDNA mutations and neuroinflammatory processes (Roubertoux et al., 2003; Stavropoulos et al., 2021; Vivian et al., 2018).

Research findings have established a robust correlation between mtDNA mutations and neuroinflammation (Corral-Debrinski et al., 1994; Grazina et al., 2006; Yu et al., 2008; Peggion et al., 2024; Risi et al., 2025). Corral-Debrinski et al. studied mtDNA mutations and their effect on AD. As a result, the mtDNA4977 deletion and mtDNA7436-np deletions were analyzed in the cortex, putamen, and cerebellum, which revealed a considerable increase in its quantity in aged individuals, suggesting that mtDNA deletions are crucial biomarkers for age-related NDDs (Corral-Debrinski et al., 1994). A clinical trial performed in the same laboratory compared levels of mtDNA deletion in patients with AD and controls, identifying significantly higher deletion levels in the cortex of patients with AD than in controls; however, the cerebellum deletion levels were similar. Deletion levels in the brains of patients in the control group increased after 75 years of age. In contrast, patients with AD had high initial levels that decreased by 80 years of age, with younger patients exhibiting more deletions than older ones (Corral-Debrinski et al., 1994). A recent study showed that the mtDNA mutations nt13708A and T4216C considerably increase the susceptibility to MS, shedding light on the role of these mutations in disease development. These mutations impair mitochondrial function in immune cells, amplifying inflammatory responses and potentially predisposing individuals to MS (Yu et al., 2008). Sometimes the acquired somatic mutations in mtDNA preferably accumulate in the brain of the aged people and play a significant role in causing NDDs. However, researchers have identified a thymine-to-guanine transition at the mtDNA414^th^ position in fibroblast of the elderly, but it was not seen in the brain of elderly patients of AD, PD, MSA, and their respective controls too. Sequencing of mtDNA fragments from elderly subjects and the clones of those mtDNA fragments revealed the absence of the 414 mutations, although occasional variations were observed. Blood samples and fibroblasts from elderly individuals also lacked this mutation. These results imply that while individual-acquired mtDNA mutations are rare, the additive burden of multiple mutations may affect mitochondrial function in the brain (Simon et al., 2001). Coskun et al. noted a higher number of occasional alterations in the mtDNA control region in patients with AD than in controls, and some of these (e.g., T414G, T414C, and T477C) were unique to those with AD (Coskun et al., 2004).

#### 4.1.2 Defects in mitochondrial fusion and fission machinery

Mitochondrial behavior and dynamics preserve functionality and ensure organelle quality control. Fusion merges individual mitochondria to create interconnected networks, whereas fission divides them into smaller fragments. The balance of these opposing mitochondrial dynamic processes is orchestrated by a network of molecular machinery, including OPA1, MFN1, and MFN2, which are responsible for fusion, and Drp1, which is responsible for fission. This network converges in a meticulous interplay that governs mitochondrial morphology, subcellular distribution, and cellular function. Disrupted mitochondrial dynamics in NDDs can impair energy production and aggravate oxidative stress, triggering neuroinflammation and worsening neuronal damage and cognitive decline. In their study, Hirai et al. observed that neurons from AD tissue displayed structural damage in their mitochondria, including shattered inner membranes and breakdown of internal architecture. Quantitative analysis revealed an overall reduction in mitochondrial quantity but a greater count in AD-affected neurons (Hirai et al., 2001). In a separate study investigating the problem with mitochondrial structure, the brains of Mfn2 KO mice had a more rounded and larger mitochondrial structure because of excessive fission and uneven fusion. This disruption caused the cellular powerhouses to break apart, resulting in a rare nerve disorder called Charcot–Marie–Tooth type 2A (Chen et al., 2007). Likewise, studies have uncovered reduced levels of mitochondrial fusion-promoting proteins OPA1, Mfn1, and Mfn2 and diminished protein production in AD-affected neurons. Concurrently, levels of Fis1, a protein that encourages mitochondrial division, are elevated. These alterations in fusion–fission equilibrium have been detected in brain cells affected by AD (Manczak et al., 2011; Wang et al., 2009). DLP1 is another major regulator of mitochondrial fission, and its low expression has been demonstrated in patients with AD (Smirnova et al., 2001; Wang et al., 2008). Upregulation of the Opa1 mitochondrial protein enhances respiratory chain efficacy and protects the tissues, offering a promising approach to addressing mitochondrial dysfunction. Increased Opa1 levels enhanced mitochondrial and motor function in two animal models with mitochondrial bioenergetic issues. These improvements included the cristae structure, respiration, motor performance, and lifespan. When Opa1 transgenic mice were crossed with muscle-specific Cox15 knockout mice, the cristae structure, breathing, motor performance, and lifespan increased. These findings suggest that Opa1 overexpression can ameliorate mitochondrial diseases by refining the cristae shape and function (Civiletto et al., 2015). Nine genes, namely, TP53, SOD2, CDKN2A, MFN2, DNM1L, OPA1, FIS1, BNIP3, and GAPDH, involved in specific mitochondrial functions have been identified as potential candidates associated with neurodegenerative processes. These genes play a role in sustaining mitochondrial homeostasis and functionality, and alterations in their expression or activity can influence the development of neurodegenerative conditions. Several studies have documented that modifications in the expression of genes regulating mitochondrial fusion, such as OPA1, MFN1, MFN2, and DLP1, also control mitochondrial fission. A subset of these genes play critical roles in neuroinflammation, directly or indirectly, by influencing mitochondrial function, oxidative stress, and cell death (Castora et al., 2022).

#### 4.1.3 Impairments in mitophagy pathways

The impaired function of the parkin and PINK1-mediated mitophagy pathways, which regulate the targeted elimination of impaired mitochondria, is another mitochondrial dysfunction. The study by Narendra et al. highlighted the significance of the PINK1-parkin pathway in mitophagy regulation. These proteins regulate mitophagy by tagging damaged mitochondria with ubiquitin molecules, preparing them for further breakdown via mitophagy. Inefficient clearance leads to a harmful accumulation of damaged mitochondria (Narendra et al., 2012). During mitochondrial depolarization, the protein PINK1 gathers on the OMM, bringing in parkin, an E3 ubiquitin ligase, further connecting ubiquitin molecules to numerous proteins. These proteins include VDAC1, Mfn1, Mfn2, and MAF, the mitochondrial assembly regulatory factor. All these proteins are found on the OMM. This process activates autophagy receptors such as p62, NBR1, and OPN, tagging mitochondrial proteins linked to the autophagy system. The linking process assists in engulfing impaired mitochondria by autophagosomes (Pickrell and Youle, 2015). However, when this pathway is not adequately regulated, it can hinder the clearance of impaired mitochondria, exacerbating mitochondrial dysfunction. When the CSF and serum samples of 60 patients with MS were analyzed to examine autophagy and mitophagy, significant differences were observed in ATG5, parkin proteins, and lactate levels between patients with and without signs of disease activity on MRI. These findings show that higher levels of autophagy and mitophagy markers, as well as lactate, are linked to the active phases of MS (Castellazzi et al., 2019). Revolutionary research on PD has identified that loss of PINK1/parkin activity, leads to significant mitochondrial damage and dopaminergic neuron death. Furthermore, mitochondria are located inside autophagosomes in the neurons of individuals with PD, implying a potential association among autophagy, mitochondrial impairment, and PD progression (Barodia et al., 2019; Bus et al., 2020; Exner et al., 2007). In a study, autosomal recessive juvenile Parkinsonism was linked to chromosome 6 and was observed to be specifically caused by deletions in the parkin gene (Kitada et al., 1998). Decreased PARK2 function led to the accumulation of mitochondria and PINK1, impairing mitophagy. Overexpressing PARK2 in these cells improved mitophagy, reducing protein buildup and restoring mitochondrial function, with similar alterations found in hippocampal samples of patients with early-stage AD (Martin-Maestro et al., 2016). These findings suggest that focusing on the regulators of mitophagy machinery could offer a promising avenue for treating neurological ailments.

### 4.2 Neuroinflammation in mitochondrial dysfunction

**FIGURE 4:**
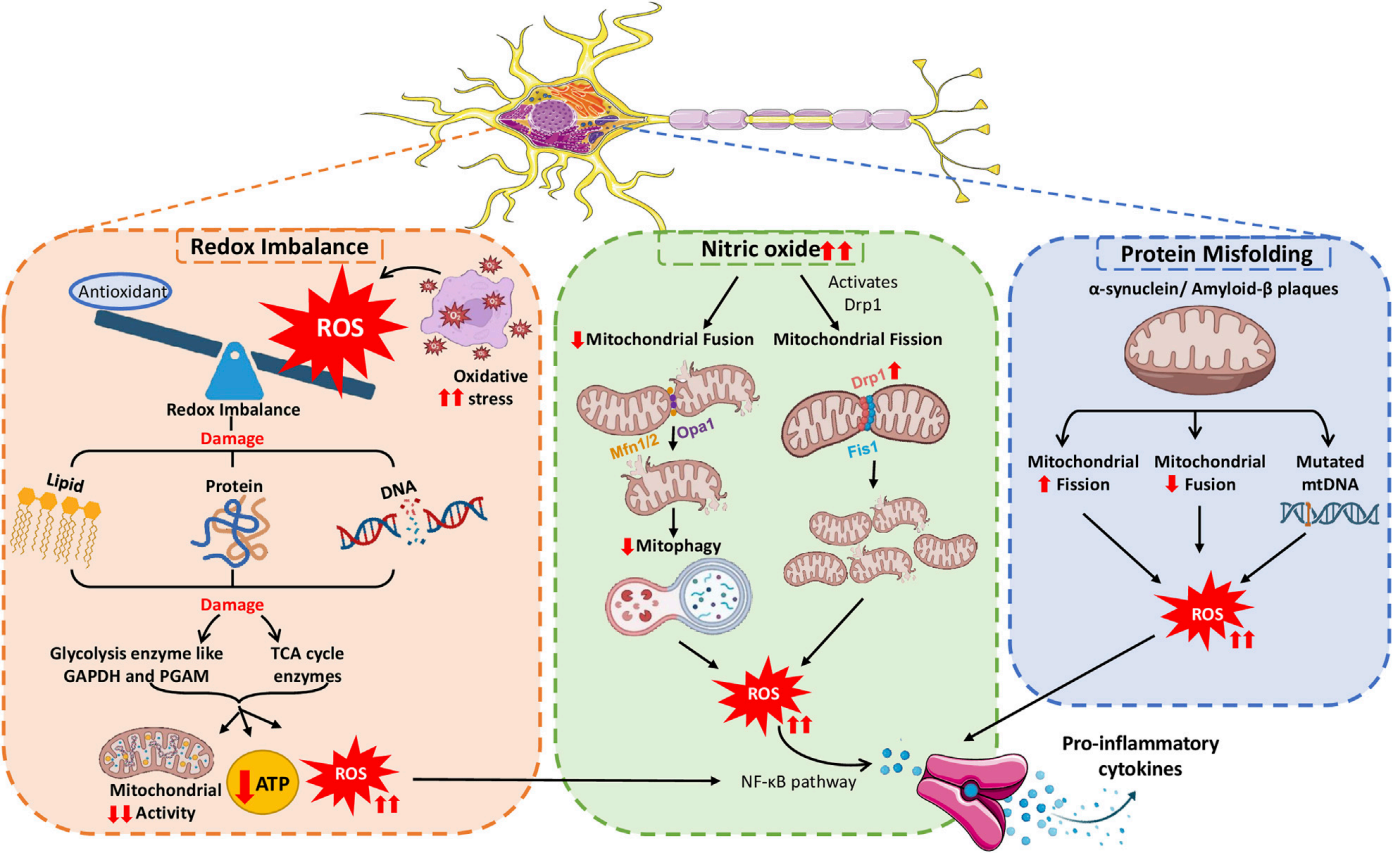
Neuroinflammation causing mitochondrial malfunction, prompting the severity of inflammatory responses.

Similar to mitochondrial dysfunction leading to neuroinflammation, neuroinflammation also affects mitochondrial function and increases the severity of the phenotype in various neurological conditions, as illustrated in Figure 4.

#### 4.2.1 Neuroinflammation-induced redox imbalance

Neurons require considerable energy for signal transmission and information processing tasks, underscoring the critical role of tightly controlled mitochondrial metabolism. Neurotransmission, ion gradient maintenance, synaptic vesicle mobilization, and synaptic plasticity rely on mitochondrial ATP production (Rangaraju et al., 2019b). ROS produced during neuroinflammation affect mitochondrial function owing to their high reactivity (Zadori et al., 2012), causing oxidative stress to mitochondria. This stress triggers microglial and astrocyte activation, releasing reactive nitrogen species (RNS) and ROS for protection against threat or damage. The accumulation of ROS and RNS interferes with redox regulation pathways by altering the function of redox–sensitive proteins and enzymes, leading to aberrant cellular responses (Uttara et al., 2009). The subsequent oxidative damage affects various mitochondrial components, impairing the organelle function. A study by Motori et al. confirmed that astrocytes respond to proinflammatory stimuli by experiencing a specific and temporary alteration in their mitochondrial dynamics, preferring fission over fusion. Motori et al. demonstrated excessive ROS generation and autophagy during this temporary stage of mitochondrial modification (Motori et al., 2013).

The inflammatory signals and NADPH oxidase produced by neuroinflammation disturb the balance of ROS production. Ultimately, this disturbance results in mitochondrial malfunctioning and reduced neuronal ATP production, worsening NDDs (Smith et al., 2022). Typically, antioxidant systems are present in the mitochondria to neutralize these ROS and maintain redox balance. Antioxidants are crucial for shielding neurons from ROS-induced oxidative damage (Butterfield et al., 2006; Thomas et al., 2022; Barnham et al., 2004). In AD, oxidative stress induces aberrant oxidative modifications in brain proteins, such as carbonic anhydrase II, disrupting cellular pH homeostasis and enzymatic activities, impairing mitochondrial function, and promoting protein aggregation (Sultana et al., 2009). The presence of protein oxidation products in the brains of individuals diagnosed with mild cognitive impairment, initial stages of AD, and advanced stages of AD indicates that oxidative pathways are gradually initiated throughout the progression of the disease (Sultana and Butterfield, 2024).

In the absence of antioxidants, ROS accumulation results in oxidative challenge and harm to neurons, contributing to NDDs. The high ROS level deteriorates, ETC., components due to increased oxidative stress and an imbalance in redox reactions within the mitochondria. This deterioration lowers ATP production further and worsens the disease. Research suggests that oxidative and redox imbalance disrupts synapses in AD (Kamat et al., 2016; Tonnies and Trushina, 2017). This disruption encompasses elevated levels of oxidative degradation of lipids, oxidation of proteins and DNA, and diminished brain glucose metabolism (Guo et al., 2013). This finding highlights the importance of maintaining the equilibrium of redox reactions in mitochondria to support optimal neuronal function and overall brain wellbeing. At the molecular level, alterations in the redox status primarily initiate neuroinflammation. Microglia produce ROS when activated by internal or external signals followed by their release into the surrounding environment. By neutralizing ROS, antioxidants help preserve mitochondrial integrity and ensure proper cellular function in the brain. Focusing on Nrf2-mediated antioxidant defense appears promising in alleviating oxidative stress-related damage in neurodegenerative conditions (Ashok et al., 2022).

Excessive ROS production can upset the redox balance inside the cells, resulting in an elevated expression of genes that cause inflammation by acting as second messengers. Consequently, when microglia become abnormally stimulated, they secrete various potentially harmful substances, including ROS, inflammatory signaling molecules, complement system components, and enzymes that break down proteins, causing tissue damage and cerebral inflammation. These changes create a long-lasting inflammatory environment that initiates or continues neurodegenerative processes (Simpson and Oliver, 2020). Nunomura et al. observed elevated levels of oxidative damage markers, including 8-hydroxyguanosine (8-OHG) and nitrotyrosine, within the neurons of patients with AD. This phenomenon emphasizes the intricate interaction between oxidative damage and the underlying disease processes that drive the onset of AD (Nunomura et al., 2001). Patients with AD exhibit considerably enhanced oxidative stress to mtDNA than individuals of similar age without the disease. The mtDNA analysis in the parietal cortex revealed a threefold increase in the levels of the oxidized nucleoside 8-OHdG in patients with AD. This finding indicates that mtDNA is vulnerable to oxidative stress-induced damage and supports the hypothesis of increased oxidative DNA damage in AD (Mecocci et al., 1994). A study by Guzman et al. revealed that normal pacemaking activity of dopaminergic neurons in SNc activates L-type calcium channels, increasing mitochondrial stress in these neurons. This stress is linked to PD. Only uncoupling proteins, not cyclophilin D, impact the slight decrease in mitochondrial membrane potential caused by this stress. Eliminating the *DJ-1* gene associated with PD development at an early age intensifies the oxidation process and decreases the expression of uncoupling proteins (Guzman et al., 2010). Furthermore, a study analyzing oxidised proteins has demonstrated that the oxidation of mitochondrial proteins affects the brain in individuals with AD (Tramutola et al., 2017). Oxidized brain proteins, particularly glycolytic and tricarboxylic acid cycle enzymes, are a distinguished feature of AD that leads to decreased ATP production. The reduced ATP levels caused by oxidized proteins significantly impact various functions in the AD-affected brain (Tramutola et al., 2017).

#### 4.2.2 Toxin-induced neuroinflammation and mitochondrial dysfunction

Lipopolysaccharide (LPS) injection models are effective tools for evaluating potential disturbances in mitochondrial function and understanding the contribution of inflammation in PD development. Striatum-specific LPS injection-initiated changes in the mitochondrial respiratory chain during an investigation. Elevated concentrations of specific biomarkers demonstrated the presence of oxidative stress. These biomarkers included protein carbonyls, lipid peroxidation products such as 4-hydroxynonenal, and indicators of protein nitration such as 3-nitrotyrosine. Moreover, the administration of LPS altered the morphology of mitochondrial inner membrane folds, posing difficulties in energy generation and causing striatum neuronal loss. These results suggest that neuroinflammation caused by LPS injection can hinder mitochondrial functioning, produce oxidative imbalance, and ultimately cause nervous system degeneration, resembling certain pathological features observed in PD (Hunter et al., 2017).

Studies have also reported a significant alteration in the mitochondrial complex (due to S-nitrosylation and nitration) of dopamine-producing neurons, which was induced for degeneration owing to LPS injection into the striatum (Choi et al., 2009). Supporting this observation, inhibition of the inducible nitric oxide synthase (iNOS) with L-N6-(1-iminoethyl)-lysine showed the alleviation in mitochondrial damage and dopaminergic cell death, caused by LPS injection. Therefore, it can be inferred that NO production by iNOS is linked to alterations in mitochondrial function in this experimental model. Voloboueva et al. demonstrated that increasing the expression of Grp75, a mitochondrial chaperone protein in LPS-stimulated BV-2 microglial cells, induced inflammation, resulting in reduced mitochondrial ROS production, sustained mitochondrial function, and improved lactate production. These findings signify that regulating inflammation by specifically targeting Grp75 can preserve mitochondrial form and function (Voloboueva et al., 2013). Studies utilizing both toxin-induced and transgenic animal models have indicated that neuroinflammatory processes substantially impact mitochondrial dysfunction. Furthermore, investigations using toxin-based models, specifically 1-methyl-4-phenyl-1,2,3,6-tetrahydropyridine (MPTP) and rotenone, have shown that an overabundance of α-synuclein expression increases neuronal susceptibility to mitochondrial toxins. For example, exposure of mutant α-synuclein transgenic mice to MPTP heightened the deterioration of dopamine-producing neurons, accompanied by an increased accumulation of α-synuclein protein clusters and mitochondrial irregularities (Song et al., 2004). In addition, administering MPTP to nonhuman primates produced eosinophilic inclusions resembling Lewy bodies, suggesting a potential association between α-synuclein accumulation and mitochondrial impairment (Kowall et al., 2000). Remarkably, mice lacking α-synuclein exhibited resilience against the damaging effects of MPTP and other toxins, suggesting that α-synuclein is a downstream effect of toxin-induced impaired mitochondrial function (Thomas et al., 2011). In a study by Calkins et al., aging-associated impairments in mitochondrial dynamics were observed in specific neuronal populations using the 3×Tg-AD mouse model, marked by an imbalance in fusion–fission equilibrium (Calkins et al., 2011).

#### 4.2.3 Neuroinflammation and altered mitochondrial dynamics

Under typical physiological conditions, mitochondrial fission and fusion events are in a delicate balance. However, neuroinflammation can disrupt this balance, hinder proper mitochondrial dynamics and compromise the neural network (Detmer and Chan, 2007; Twig et al., 2008). NO has been reported to control the activity of DRP1 and the process of mitochondrial fission. For instance, Motori et al. found that inflammation caused by NO triggers the activation of DRP1, resulting in the fragmentation of astrocyte mitochondria. The fragmented mitochondria produce excessive ROS and generate insufficient ATP, leading to NF-κB pathway activation and proinflammatory cytokine secretion (Motori et al., 2013). Moreover, inflammation in the brain enhances the phosphorylation of DRP1 at serine 616, stimulating uncontrolled mitochondrial fragmentation (Rahman and Suk, 2020). An autopsy study of individuals with AD, revealed that as the disease progresses, mitochondrial fusion proteins OPA1, Mfn1, and Mfn2 decrease, and fission proteins DRP1 and Fis1 increase. This study further examined the effect of high levels of the inflammatory mediator IL-1β on regulating the division and merging of mitochondria in individuals with AD (Wang et al., 2009).

#### 4.2.4 Misfolded proteins causing mitochondrial dysfunction

Aβ protein accumulation in AD impairs cellular energy generation and increases the levels of harmful reactive chemicals, upsetting the regular operation of cellular powerhouses and escalating brain inflammation. Similarly, α-synuclein protein buildup in PD reduces mitochondrial function by blocking an essential step in energy generation and triggering brain inflammatory reactions. In addition, Aβ aggregation affects mitochondrial electrical charge distribution, division and fusion capabilities, and energy-generating systems. Furthermore, this aggregation modifies mitochondrial genetic makeup, further impairing their capacity for efficient operation (Swerdlow, 2018). In an animal model of AD, Calkins et al. examined primary neuronal cells from Tg2576 mice (expressing the amyloid precursor protein) and observed that oligomeric Aβ protein disrupted synapses and mitochondria. This disruption caused mitochondria to move less and undergo more fission, ultimately damaging their structure. These neurons exhibited higher Aβ accumulation and apoptotic death than wild-type neurons, illustrating that Aβ buildup causes mitochondrial and synaptic dysfunction, culminating in neurodegeneration (Calkins et al., 2011). Another research group performed experiments using the Tg2576 model to study the gene expression pattern in three different phases of the disease: early stage (2 months), pre-plaque stage (5 months), and post-plaque stage (18 months). The findings revealed that genes related to apoptosis and mitochondrial energy metabolism were turned on in all three stages. Using Northern blot analysis, researchers confirmed that neurons overexpressing mitochondrial genes, such as *ATPase-6*, exhibited oxidative damage (Reddy et al., 2004). Moreover, in a mouse model, mitochondrial accumulation of α-synuclein impaired their function by reducing complex I activity and increasing neuroinflammation. Studies have observed that a specific segment of α-synuclein targets mitochondria, disrupting complex I function. This interaction leads to elevated production of ROS and potentially contributes to cellular stress and damage. The considerable accumulation of α-synuclein and the reduced activity of mitochondrial complex I in the striatum and substantia nigra of affected brains imply that α-synuclein localized to mitochondria suppresses the function of this crucial energy-producing enzyme complex (Devi et al., 2008).

Signaling molecules released by activated immune cells can hinder the functioning of mitochondria in the brain, alluding that inflammation-induced mitochondrial impairment is crucial in aggravating nerve cell degeneration and decline. For example, a study on a PD mouse model found that mitochondrial damage in microglia caused them to produce excessive proinflammatory cytokines, exacerbating the neuroinflammatory response. This observation highlights the indirect influence of mitochondrial health on regulating microglial inflammation. Moreover, a study on patients with PD identified a correlation between increased serum cytokine levels and the severity of cognitive and motor symptoms. Increased proinflammatory cytokines in such patients were associated with cognitive deterioration and accelerated motor decline, whereas decreased levels led to better outcomes (Fu et al., 2023).

## 5 Therapeutic strategies targeting mitochondrial dysfunction/neuroinflammation

Recent studies have shown that the initiation of neuroinflammatory processes is considerably influenced by mitochondrial dysfunction (Cai and Tammineni, 2017; Calkins et al., 2011; Cheng et al., 2010; Chistiakov et al., 2014; Dmytriv et al., 2023). As sterile inflammation is involved in the body’s natural defensive processes, it is being studied as a possible therapeutic target. Sterile inflammation is a prevalent feature in NDDs. The possibility of targeting DAMPs is highlighted by the likelihood that the uncontrollable release of DAMPs and the ensuing overactivation of PRRs produce neuroinflammation, and the molecules involved in their signaling pathways. Suppressing mitophagy is associated with a reduced lifespan, whereas enhancing the process can prolong the lifespan (Picca et al., 2020).

**FIGURE 5:**
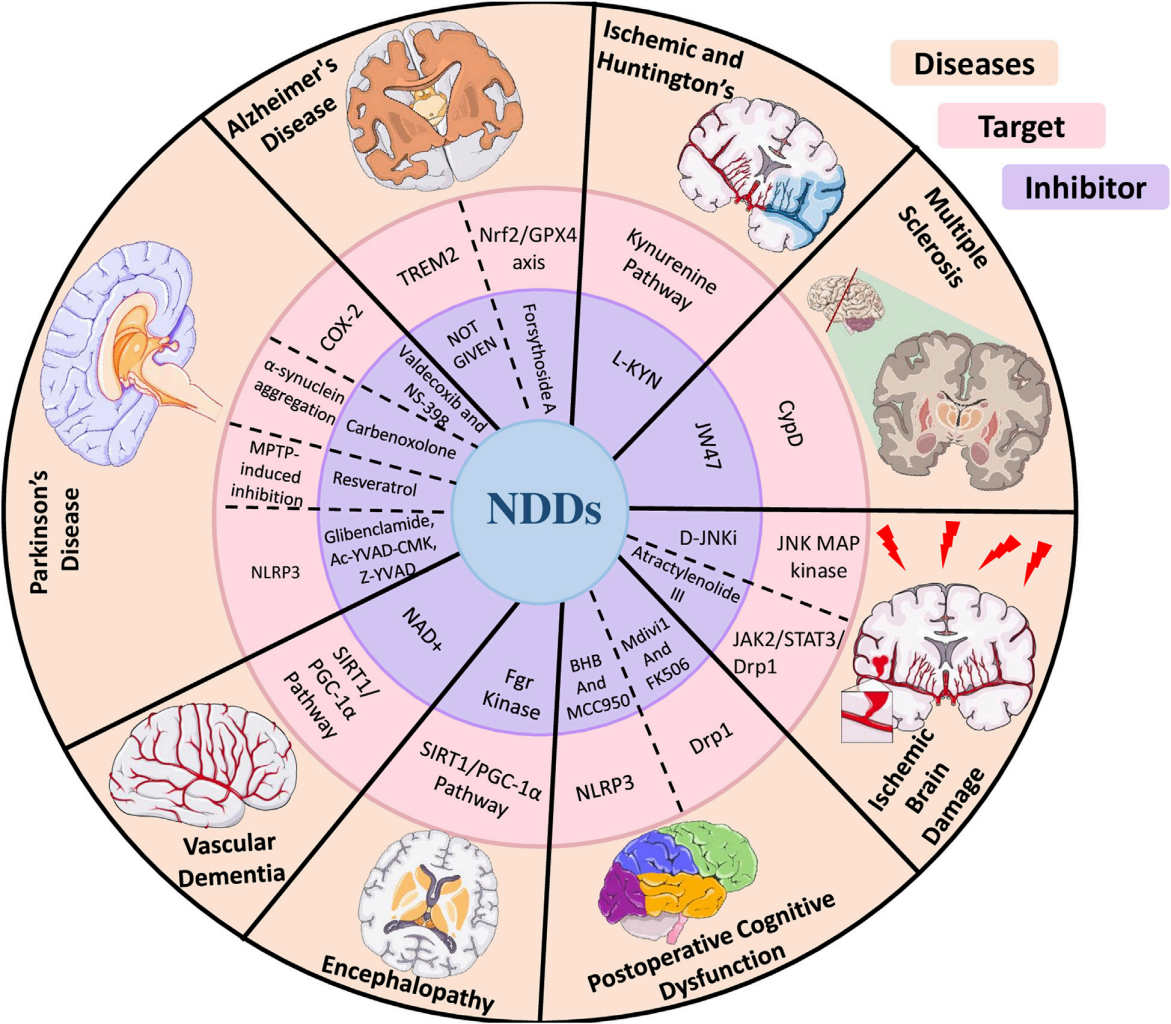
Diagram illustrating the impact of various inhibitors on neuroinflammation and mitochondrial dysfunction across multiple neurological disorders.

Researchers are investigating various approaches to combat mitochondrial dysfunction, specifically in the context of neuroinflammation (Table 1, Figure 5). Treatment with NAD^+^ has been reported to exhibit anti-inflammatory and antioxidant properties in controlling oxidative stress. A study found that NAD^+^ administration enhanced cognitive ability and decreased neuroinflammation in an animal model with reduced cerebral blood flow. This finding could be attributed to protective mechanisms aimed at maintaining optimal mitochondrial function. NAD^+^ also protects against neuroinflammation, mitochondrial dysfunction, and minimizes the hypoxia-induced oxidative harm by activating the Sirt1/PGC-1α signaling pathway (Zhao et al., 2021). Although NAD^+^ administration has shown potential in decreasing neuroinflammation and preserving mitochondrial function, an alternative strategy for addressing α-synuclein aggregation, a significant factor in PD, is the utilization of carbenoxolone (Cbx). α-Synuclein aggregation, neuroinflammation, and mitochondrial dysfunction contribute to PD, and treatment with Cbx decreases the formation of α-synuclein clumps, resulting in reduced oxidative stress and enhanced mitochondrial function (Thakur and Nehru, 2015). Furthermore, α-synuclein plays a role in neuroinflammation related to PD (Han and Le, 2023). Moving on to AD, Ferroptosis and neuroinflammation were examined in a study focusing on forsythoside A, a component of *Forsythia suspensa*, which improves mitochondrial function in Aβ1-42-exposed N2a cells, reducing proinflammatory factors in LPS-stimulated BV2 cells (Wang et al., 2022). TREM2, another important factor, is a transmembrane receptor in myeloid cells and is linked to AD. Its variants, which cause partial loss of function and alter microglial behavior, can triple the risk of AD (Carmona et al., 2018). In addition, the neuroprotective properties of specific COX-2 inhibitors, such as valdecoxib and NS-398, have been proven in mice with MPTP-induced neurotoxicity (Gupta et al., 2011). Similarly, resveratrol, a natural polyphenol, has mitigated MPTP-triggered toxicity in *Drosophila melanogaster* (Abolaji et al., 2018).

**TABLE 1:** Different inhibitors show their effect on neuroinflammation and mitochondrial dysfunction in various neurological disorders.

S. No.	Inhibitor	Target	Disease	Reference
1	Valdecoxib and NS-398	Cyclooxygenase (COX)-2	PD	Gupta et al. (2011)
2	D-JNKi	JNK MAP kinase	Ischemic Brain Damage	Nijboer et al. (2013)
3	Carbenoxolone	α-synuclein aggregation	PD	Thakur and Nehru (2015)
4	JW47	CypD	MS	Warne et al. (2016)
5	Resveratrol	MPTP-induced inhibition of AChE activity, accumulations of hydrogen peroxide	PD	Abolaji et al. (2018)
6	NOT GIVEN	TREM2	AD	Carmona et al. (2018)
7	MCC950 and BHB	NLRP3 inflammasome	POCD	Wei et al. (2019)
8	Atractylenolide III	JAK2/STAT3/Drp1	Ischemic Brain Damage	(Zhou et al., 2019)
9	Nicotinamide adenine dinucleotide (NAD+)	Sirt1/PGC-1α pathway	Vascular Dementia	Zhao et al. (2021)
10	Mdivi-1 and FK506	Drp1	POCD	Yang et al. (2022)
11	Forsythoside A	Nrf2/GPX4 axis	AD	Wang et al. (2022)
12	Fgr kinase	SIRT1/PGC-1α pathway	Encephalopathy	Liu et al. (2023)
13	MCC950, Glibenclamide, Ac-YVAD-CMK, Z-YVAD, VX765, Anakinra, Mdivi-1, Perillyl alcohol, Urolithin A, GW501516, Melatonin, NIM811	NLRP3 inflammasome	PD	Han and Le (2023)

Researchers have documented that blocking Fgr, a type of tyrosine kinase, can improve survival rates, reduce neuroinflammation, and alleviate cognitive dysfunction, oxidative stress, and mitochondrial malfunction in sepsis-associated encephalopathy. The interaction between Fgr and SIRT1, which leads to the activation of SIRT1/PGC-1α, enhances the protective properties of the Fgr inhibitor (Guzman et al., 2010). NDDs are linked to mitochondrial malfunction, which results in reduced ATP generation and increased oxidative damage. Both acute and chronic diseases are characterized by kynurenine pathway dysregulation. This pathway includes 3-hydroxy-L-kynurenine, which induces toxicity via free radicals, and quinolinic acid, a potent neurotoxin. L-kynurenine possesses vasoactive properties and may increase kynurenic acid levels in the brain. Despite being neuroprotective, kynurenic acid should not be administered systemically to humans because of its pharmacokinetic properties (Tramutola et al., 2017). One disorder linked to neuroinflammation is postoperative cognitive dysfunction (POCD). An approach to address this issue is to target mitochondrial ROS and the NLRP3 inflammasome, which may alleviate neuroinflammation in patients with POCD. It is a typical neurological aftereffect of general anesthesia and surgery. Hippocampal IL-1β expression is upregulated in response to surgery-induced systemic inflammation. Studies have reported that mitochondria-generated ROS influence IL-1β expression via the NLRP3 inflammasome (Wei et al., 2019).

A study has explored the impacts of a tractylenolide III (A III) on neuroinflammation and mitochondrial homeostasis in microglia, specifically with regard to stroke. The findings showed promising results in reducing brain damage and regulating cytokine expression. Nevertheless, the precise effect of A III on neuroinflammation and the maintenance of mitochondrial homeostasis in microglia after a stroke is yet to be elucidated. Therapy with A III and AG490, a JAK2 inhibitor, has been shown to reduce brain damage, restore cerebral blood flow, and decrease expressions of proinflammatory cytokines in MCAO mice and primary microglia, activated by oxygen–glucose deprivation (Zhou et al., 2019).

JW47, a newly developed inhibitor of cyclophilin D (CypD), has shown promise in specifically targeting the mitochondrial permeability transition pore for treating NDDs. CypD, a positive regulator of this pore, is a key target for drugs used to treat neurodegenerative conditions. The lack of specificity and unintended effects of existing inhibitors have prompted the development of the novel JW47, designed exclusively to target and inhibit CypD. This new inhibitor has shown encouraging outcomes. JW47 exhibited targeted suppression of CypD in cells and lower toxicity than cyclosporine (Warne et al., 2016). In neonatal encephalopathy, the JNK MAP kinase inhibitor D-JNKi exerted protective effects against ischemic brain injury and impairments related to cognition and movement by preserving mitochondrial integrity and alleviating neuroinflammation. Few therapeutic options are available for neonatal encephalopathy, which is associated with a high death rate and developmental difficulties. A JNK MAP kinase inhibitor called D-JNKi was evaluated for its effects on neuroinflammation and neuronal injury in a model of newborn rats. This treatment prevented the phosphorylation of nuclear c-Jun, reducing cerebral cytokine production and the activity of the AP-1 transcription factor. Lipid peroxidation and ATP loss were consequently halted, maintaining mitochondrial integrity. D-JNKi treatment offered protection against cognitive and motor impairment and ischemic brain injury (Nijboer et al., 2013).

Excessive oxidative stress is responsible for the development of NDDs linked to mitochondrial impairment. Researchers have explored antioxidants as a possible treatment strategy for alleviating oxidative stress and enhancing cell survival in these conditions. Coenzyme Q10 and its analogs, such as idebenone, have been examined for their antioxidant properties and ability to improve mitochondrial function in NDDs (Glover et al., 2010). The European Medicines Agency has approved Idebenone to treat Leber hereditary optic neuropathy (LHON), a mitochondrial disease affecting the optic nerve. EPI-743 (PTC-743), another antioxidant compound, has shown promise in treating Leigh syndrome, a neurological illness that progresses over time. Furthermore, this compound can potentially reverse vision loss in patients with LHON and reduce seizures in children with RARS2 deficiency, a mitochondrial syndrome associated with epilepsy (Hirano et al., 2018; Klopstock et al., 2013). Elamipretide, a mitochondria-targeted tetrapeptide, has been investigated in NDDs such as LHON, but the results are yet to be published (Szeto, 2014). Sonlicromanol (KH176), an orally bioavailable antioxidant and redox modulator, has been studied in patients carrying a specific mitochondrial mutation (m.3243A>G) related to neurological symptoms, showing tolerability but limited efficacy (Janssen et al., 2019). Dysregulation of mitophagy is associated with several age-related diseases. Numerous clinical trials are ongoing to assess the efficacy of drugs that modulate mitophagy in treating age-related diseases/NDDs. A clinical trial (NCT02472340) was conducted to determine the levels of mitophagy and autophagy in the muscle tissue of healthy and pre-frail elderly individuals. Mitophagy inducers such as urolithin A (Andreux et al., 2019), rapamycin analogs (Sage-Schwaede et al., 2019), and NAD^+^ precursors have shown potential in animal and human investigations to eliminate damaged mitochondria, delay cellular senescence, and extend lifespan. Although mitophagy inducers have shown promise, further research is needed to establish their clinical efficacy. Combining mitophagy enhancers with mitochondrial biogenesis activators may offer a comprehensive approach to treating mitochondrial dysfunction.

Clinical studies focusing on mitochondrial targets should examine interventions such as antioxidant therapies, manipulation of mitochondrial dynamics, and augmentation of mitochondrial biogenesis and functions. These aspects are presently being evaluated in clinical trials to assess their safety, effectiveness, and potential as treatments for neurological disorders characterized by neuroinflammation and mitochondrial dysfunction.

## 6 Conclusion

There has been a significant change in our understanding of NDDs in the past few years, from the belief that they were primarily disorders of protein homeostasis characterized by abnormal protein accumulation that caused cell damage. It is now evident that numerous other processes simultaneously act as initiator elements. Recent research has revealed that several of these processes are correlated with dysbalanced mitochondrial function, supporting the notion that inflammation in the brain is the primary pathogenic pathway in nondiagnostic depressions. Neuroinflammation is mainly caused by the redox state at the molecular level. Both ROS production and the response to cellular alterations caused by ROS are performed by mitochondria. As a result, neuroinflammation can both cause and be caused by mitochondrial dysfunction.

Therefore, inflammatory responses in neuronal cells and mitochondrial dysfunction are closely related variables that affect each other as the disease progresses. Damaged mitochondria can accumulate and trigger a destructive cycle of inflammation that causes NLRP3 inflammasome-dependent inflammation in microglia. Damaged neurons can release DAMPs, such as mtDNA, into the extracellular space, exacerbating inflammation. Structural aberrations in the number, form, and turnover of mitochondria inside the cell have offered profound insights. In addition, the resolution of neuroinflammation and the preservation of homeostasis may be associated with mitochondria.

In PD models, mitophagy induces the production of IL-10, which regulates inflammation. More intriguingly, damaged mitochondria are discharged to prevent excessive ROS production, as seen during stroke, and astrocytes stimulated by inflammation may actively move mitochondria to adjacent neurons to alleviate or avoid tissue damage. A clear picture of how mitochondria function in neuroinflammation and neurodegeneration is emerging. Although several NDDs share mitochondrial dysfunction as a common trait, our understanding of how it functions is becoming increasingly comprehensive.

In conclusion, studies aimed at addressing mitochondrial dysfunction in clinical settings have the potential to treat neuroinflammation associated with NDDs. Owing to the intricate nature of mitochondrial biology in these disorders, a multimodal therapeutic strategy is needed, probably involving innovative pharmacological treatments, nutraceuticals, and epigenetic modulators. More investigations are required to determine the best targets and therapeutic approaches in this field.
